# Antigenicity and Protective Efficacy of a *Leishmania* Amastigote-specific Protein, Member of the Super-oxygenase Family, against Visceral Leishmaniasis

**DOI:** 10.1371/journal.pntd.0002148

**Published:** 2013-03-28

**Authors:** Vivian T. Martins, Miguel A. Chávez-Fumagalli, Lourena E. Costa, Adriana M. C. C. Martins, Paula S. Lage, Daniela P. Lage, Mariana C. Duarte, Diogo G. Valadares, Rubens D. M. Magalhães, Tatiana G. Ribeiro, Ronaldo A. P. Nagem, Wanderson D. DaRocha, Wiliam C. B. Régis, Manuel Soto, Eduardo A. F. Coelho, Ana Paula Fernandes, Carlos A. P. Tavares

**Affiliations:** 1 Departamento de Bioquímica e Imunologia, Instituto de Ciências Biológicas, Universidade Federal de Minas Gerais, Belo Horizonte, Minas Gerais, Brazil; 2 Programa de Pós-Graduação em Ciências da Saúde: Infectologia e Medicina Tropical, Faculdade de Medicina, Universidade Federal de Minas Gerais, Belo Horizonte, Minas Gerais, Brazil; 3 Programa de Pós-Graduação em Ciências Farmacêuticas, Faculdade de Farmácia, Universidade Federal de Minas Gerais, Belo Horizonte, Minas Gerais, Brazil; 4 Departamento de Patologia Clínica, COLTEC, Universidade Federal de Minas Gerais, Belo Horizonte, Minas Gerais, Brazil; 5 Departamento de Bioquímica, Universidade Federal do Paraná, Curitiba, Brazil; 6 PUC Minas and Minasfungi do Brasil LTDA, Belo Horizonte, Minas Gerais, Brazil; 7 Centro de Biología Molecular Severo Ochoa, CSIC-UAM, Departamento de Biología Molecular, Universidad Autónoma de Madrid, Madrid, Spain; 8 Departamento de Análises Clínicas e Toxicológicas, Faculdade de Farmácia, Universidade Federal de Minas Gerais, Belo Horizonte, Minas Gerais, Brazil; National Institutes of Health, United States of America

## Abstract

**Background:**

The present study aimed to evaluate a hypothetical *Leishmania* amastigote-specific protein (LiHyp1), previously identified by an immunoproteomic approach performed in *Leishmania infantum*, which showed homology to the super-oxygenase gene family, attempting to select a new candidate antigen for specific serodiagnosis, as well as to compose a vaccine against VL.

**Methodology/Principal Findings:**

The LiHyp1 DNA sequence was cloned; the recombinant protein (rLiHyp1) was purified and evaluated for its antigenicity and immunogenicity. The rLiHyp1 protein was recognized by antibodies from sera of asymptomatic and symptomatic animals with canine visceral leishmaniasis (CVL), but presented no cross-reactivity with sera of dogs vaccinated with Leish-Tec, a Brazilian commercial vaccine; with Chagas' disease or healthy animals. In addition, the immunogenicity and protective efficacy of rLiHyp1 plus saponin was evaluated in BALB/c mice challenged subcutaneously with virulent *L. infantum* promastigotes. rLiHyp1 plus saponin vaccinated mice showed a high and specific production of IFN-γ, IL-12, and GM-CSF after *in vitro* stimulation with the recombinant protein. Immunized and infected mice, as compared to the control groups (saline and saponin), showed significant reductions in the number of parasites found in the liver, spleen, bone marrow, and in the paws' draining lymph nodes. Protection was associated with an IL-12-dependent production of IFN-γ, produced mainly by CD4 T cells. In these mice, a decrease in the parasite-mediated IL-4 and IL-10 response could also be observed.

**Conclusions/Significance:**

The present study showed that this *Leishmania* oxygenase amastigote-specific protein can be used for a more sensitive and specific serodiagnosis of asymptomatic and symptomatic CVL and, when combined with a Th1-type adjuvant, can also be employ as a candidate antigen to develop vaccines against VL.

## Author Summary

Life-long immunity to leishmaniasis in recovered patients has inspired the development of vaccines against disease. The present study aimed to evaluate a non-described hypothetical *Leishmania* amastigote-specific protein, identified by an immunoproteomic approach in *L. infantum*, attempting to select a new candidate antigen for specific serodiagnosis and a vaccine against visceral leishmaniasis (VL). The recombinant protein (rLiHyp1) was recognized by antibodies from sera of asymptomatic and symptomatic canine visceral leishmaniasis (CVL), but presented no cross-reactivity with sera of vaccinated dogs, those with Chagas' disease or healthy animals. In addition, the rLiHyp1 plus saponin was able to induce a Th1 response, which was based on the production of high levels of IFN-γ, IL-12, and GM-CSF after *in vitro* stimulation in BALB/c mice. The protective efficacy of rLiHyp1 plus saponin was evaluated in mice challenged with *L. infantum* promastigotes. Challenged and vaccinated mice showed significant reductions in the number of parasites in all evaluated organs, and the protection was associated with a Th1-type response. Therefore, the present study reveals a new potential candidate for the improvement of serodiagnosis of CVL, as well as an effective vaccine candidate against VL.

## Introduction

Visceral leishmaniasis (VL) caused by *Leishmania donovani* and *L. infantum/L. chagasi* represents an important disease in the world, leading to nearly 500,000 new cases and 50,000 deaths annually. The first choice of treatment for all forms of leishmaniasis is still based on the use of the parenteral administration of pentavalent antimonials; however, increased parasite resistance and several side effects reported by patients have been important problems [1], [2]. Liposomal amphotericin B is effective but is too expensive for most patients [3]. Results from clinical trials using oral miltefosine are encouraging; however, therapy is linked to both potential toxicity and teratogenicity, and should not be given to childbearing-age women [4]. Therefore, the development of new strategies to prevent leishmaniasis has become a high priority.

The evidence of life-long immunity to leishmaniasis has inspired the development of prophylactic vaccination protocols against the disease, but few have progressed beyond the experimental stage. Most experimental vaccines have focused on the mouse model for cutaneous leishmaniasis. Several studies have demonstrated the Type-1 cells mediated immunity-dependence for protective responses against disease. Moreover, Th1 cells response has also been correlated with protection against VL [5]. In this context, the protective immunity in murine VL primarily depends on an IL-12-driven Th1 cells response, leading to an increased IL-2 and IFN-γ production. Substantial uptake of inducible NO syntase by IFN-γ generates NO from splenic and liver cells, thereby controlling parasite multiplication in these organs [6], [7]. By contrast, TGF-β, IL-10, and IL-13 represent major disease promoting cytokines, leading in turn to the suppression of the Th1 response [8]. Low levels of IL-4 commonly enhance vaccine-induced protection by indirectly increasing IFN-γ production by T cells [9].

In recent decades, the majority of studies have focused on *Leishmania* promastigote antigens for vaccine development [10], [11], [12], [13]; however, amastigote antigens also seem to be appropriate targets for the immune responses elicited by vaccines, given that after a few hours of infection and during the active disease, this parasite stage becomes exposed to the host immune system [14]. In addition, the fact that promastigotes can be easily cultured *in vitro*, as opposed to axenic amastigotes, has hampered the identification of amastigote-specific stage antigens [15].

In the present work, a hypothetical amastigote-specific *L. infantum* protein, LiHyp1 (XP_0014689 41.1), which has been identified by an immunoproteomic approach, was recognized by antibodies present in sera samples of dogs with asymptomatic and symptomatic VL [16]. The amastigote-specific *Leishmania* protein gene (LiHyp1) is predicted to encode a protein with a theoretical molecular weight of 36.6 kDa. An *in silico* sequence comparison revealed that LiHyp1 belongs to the super-oxygenase family in *Leishmania*, and is an alkylated DNA repair protein. The ability of the recombinant protein (rLiHyp1) to induce protection against infection with virulent *L. infantum* promastigotes was assessed in BALB/c mice. The results showed that rLiHyp1 was antigenic and specifically recognized by canine VL (CVL) sera, whereas a Th1 response, induced by immunization of a combination of rLiHyp1 and saponin, was able to confer protection against *L. infantum*. This protection correlated with a *Leishmania* antigens-specific and IL-12-dependent IFN-γ production, mediated mainly by CD4 T cells, as well as by a diminished production of parasite-specific IL-4 and IL-10.

Thus, the present study demonstrates that this unique amastigote-specific protein, a member of the super-oxygenase family in *Leishmania*, can be a new candidate for the improvement of serodiagnosis of CVL and, when associated with a Th1-type adjuvant, to develop an effective vaccine against VL.

## Materials and Methods

### Ethics statement

Experiments were performed in compliance with the National Guidelines of the Institutional Animal Care, and Committee on the Ethical Handling of Research Animals (CEUA) from the Federal University of Minas Gerais (Law number 11.794, 2008), with code number 043/2011. Sera samples used in this study were kindly provided by Prof. Fernando Aécio de Amorim Carvalho (Department of Pharmacology and Biochemistry, UFPI) and Prof. Maria Norma Melo (Department of Parasitology, Institute of Biological Sciences, UFMG).

### Mice and parasites

Female BALB/c mice (8 weeks age) were obtained from the breeding facilities of the Department of Biochemistry and Immunology, Institute of Biological Sciences, UFMG, and were maintained under specific pathogen-free conditions. Experiments were carried out using the *L. infantum* (MOM/BR/1970/BH46) strain. Parasites were grown at 24°C in Schneider's medium (Sigma, St. Louis, MO, USA), supplemented with 10% heat-inactivated fetal bovine serum (FBS, Sigma), 20 mM L-glutamine, 200 U/mL penicillin, and 100 µg/mL streptomycin, at pH 7.4. The soluble *L. infantum* antigenic extract (SLA) was prepared from 1×10^10^ stationary-phase promastigote cultures (5–7 day-old), as described [17]. Parasites were kindly provided by Prof. Maria Norma Melo.

### Cloning of DNA sequence coding for *L. infantum* hypothetical protein, LiHyp1

The LiHyp1 (XP_001468941.1) nucleotide and amino acid sequences used in this study were obtained from the National Center for Biotechnology Information (http://www.ncbi.nlm.nih.gov). The local alignment of the LiHyp1 sequence against the available complete genomes of other organisms was performed by BLAST. Analyses of basic physical and chemical properties, as well as phylogenetic analysis, were performed in a TriTrypDB database (http://tritrypdb.org). The recombinant protein (rLiHyp1) was obtained after having cloned a DNA *L. infantum* fragment containing the LiHyp1 coding region. Initially, genomic DNA was extracted by a phenol:chloroform extraction, as described [17], and it was used as a template. *Forward* (5′-GAAGGATCCAGCATGTCTATCGTGTCGAG-3′) and *reverse* (5′-GGAAAGCTTCGCTTGCGGCGTCACGTGAGC-3′) primers were designed according to the DNA sequence of the ORF described in the *L. infantum* genome sequence database (LinJ.35.1290). The PCR product was cloned into the pGEM-T *easy* vector confirmed by sequencing and transferred to the pET21a expression vector (Novagen), using the *BamHI* and *HindIII* restriction enzymes included in the primers for this purpose (underlined). Recombinant plasmid was transformed into *Escherichia coli* BL21 (DE3). The recombinant protein expression was performed by adding 0.5 mM IPTG (isopropyl-β-D-thiogalactopyranoside, Promega, Montreal, Canada) for 4 h at 37°C, when cells were lysed by a homogenizer after five passages through the apparatus. The product was centrifuged at 13.000× *g* for 20 min at 4°C, while the rLiHyp1, containing a tag of 6× residues of histidine, was purified under non-denaturing conditions, using a 5 mL HIS-Trap column (GE Healthcare Life Science) attached to an FPLC (GE Healthcare Life Science) system. After purification, the recombinant protein was passed through a polymyxin-agarose column (Sigma) to remove residual endotoxin content.

### Canine sera and ELISA for immunodiagnosis and immunoblotting

To evaluate the antigenicity of rLiHyp1, sera samples from healthy (n = 37), vaccinated with Leish-Tec® (n = 18), *T. cruzi* experimentally infected (n = 18), asymptomatic (n = 19) and symptomatic *L. infantum*-infected dogs (n = 15) were used. All animals were considered symptomatic when three or more of the following symptoms were present: loss of weight, alopecia, adenopathy, onychogryphosis, hepatomegaly, conjunctivitis and exfoliate dermatitis on the nose, tail and ear tips; and asymptomatic when they were free from clinical symptoms. In the infected animals, the diagnosis of the disease was defined when amastigotes were seen in Giemsa stained smears of bone marrow aspirates or promastigotes were identified on culture of peripheral blood or bone marrow aspirates. Sera were considered positive when tested by indirect immunofluorescence. A titration curve was performed to determine the best protein concentration and antibody dilution to perform ELISA. Plates (Falcon) were sensitized with rLiHyp1 (1.0 µg/well) or SLA (0.5 µg/well) for 18 h at 4°C. Free binding sites were blocked with a PBS-Tween 20 0.05% (PBST) and 5% casein solution for 2 h at 37°C. After five washes with PBST, plates were incubated with 100 µL of canine sera for 1 h at 37°C. Serum samples were diluted 1∶200 in PBST and 0.5% casein. After, plates were washed seven times with PBST and incubated with 1∶10.000 anti-dog IgG antibody (Sigma, St. Louis, USA) horseradish peroxidase conjugated. The reaction was developed through incubation with H_2_O_2_, along with *orto*-phenylenediamine and citrate-phosphate buffer pH 5.0, for 30 min in the dark, and was stopped by adding H_2_O_2_ 2 N. Optical densities were read at 492 nm in an ELISA microplates spectrophotometer (Molecular Devices, Spectra Max Plus, Concord, Canada).

For immunoblotting experiments, the recombinant protein was submitted to a 10% SDS-PAGE and blotted onto a nitrocellulose membrane (0.2 µm pore size, Sigma, St. Louis, USA). Membranes were blocked with PBST and 5% casein solution, and were incubated for 2 h at 37°C before the first incubation with a pool of sera samples of asymptomatic CVL, diluted 1∶100 in PBST. Peroxidase conjugated anti-dog IgG (1∶5.000) was used as a second antibody (Sigma). Reactions were revealed by adding chloronaphtol, diaminobenzidine, and H_2_O_2_.

### Immunization and challenge infection

Mice (n = 8, per group) were vaccinated subcutaneously in their left hind footpad with 25 µg of rLiHyp1 associated with 25 µg of saponin (*Quillaja saponaria* bark saponin, Sigma), with adjuvant or only diluent (PBS). Three doses were administered at 2-week intervals. Four weeks after the final immunization, animals (n = 4, per group) were euthanized for the analysis of the immune response elicited by vaccination. At the same time, the remaining animals were infected subcutaneously in the right hind footpad, with virulent 1×10^7^ stationary-phase promastigotes of *L. infantum*, when 10 weeks after the animals were euthanized, and the liver, spleen, bone marrow (BM), and the paws' draining lymph nodes (dLN) were collected to determine parasite burden and evaluation of the immune response.

### Estimation of parasite load

The liver, spleen, BM, and dLN were collected for parasite quantification, following a limiting-dilution protocol [18]. Briefly, the organs were weighed and homogenized using a glass tissue grinder in sterile PBS. Tissue debris was removed by centrifugation at 150× *g*, and cells were concentrated by centrifugation at 2000× *g*. Pellets were resuspended in 1 mL of Schneider's insect medium supplemented with 20% FBS. Two hundred and twenty microliters were plated onto 96-well flat-bottom microtiter plates (Nunc, Nunclon®, Roskilde, Denmark) and diluted in log-fold serial dilutions in supplemented Schneider's medium with a 10^−1^ to 10^−20^ dilution. Each sample was plated in triplicate and read 7 days after the beginning of the culture at 24°C. Pipette tips were discarded after each dilution to avoid carrying adhered parasites from one well to another. Results are expressed as the negative log of the titer (*i.e.*, the dilution corresponding to the last positive well) adjusted per microgram of tissue.

### Cytokine production

Splenocyte cultures and cytokine assays were performed before infection and at 10^th^ week after challenge, as described [17]. Briefly, single-cell preparations from spleen tissue were plated in duplicate in 24-well plates (Nunc) at 5×10^6^ cells per mL. Cells were incubated in DMEM medium (non-stimulated, background control), or separately stimulated with SLA (25 µg mL^−1^) or rLiHyp1 (20 µg mL^−1^), at 37°C in 5% CO_2_ for 48 h. IFN-γ, IL-4, IL-10, IL-12, and GM-CSF levels were assessed in the supernatants by a sandwich ELISA provided in commercial kits (BD OptEIA TM set mouse IFN-γ (AN-18), IL-12 and GM-CSF; Pharmingen, San Diego, CA, USA; and Murine IL-4 and IL-10 ELISA development kits; PeproTech®, São Paulo, Brazil); following manufacturer's instructions. In order to block IL-12, CD4, and CD8 mediated T cell cytokine release, spleen cells of mice vaccinated with rLiHyp1 plus saponin and challenged with *L. infantum* were *in vitro* stimulated with SLA (25 µg mL^−1^), and incubated in the presence of 5 µg mL^−1^ of monoclonal antibodies (mAb) against mouse IL-12 (C17.8), CD4 (GK 1.5), or CD8 (53-6.7). Appropriate isotype-matched controls – rat IgG2a (R35-95) and rat IgG2b (95-1) – were employed in the assays. Antibodies (no azide/low endotoxin™) were purchased from BD (Pharmingen, San Diego, CA, USA).

### Statistical analysis

The statistical analysis was made using the GraphPad Prism software (version 5.0 for Windows). Statistical analysis with the vaccinated and/or infected mice was performed by one-way analysis of variance (ANOVA), using the Bonferroni's post-test for multiple comparisons of groups. Receiver Operating Characteristic (ROC) curves were used to analyze the data obtained using sera samples of dogs. Statistical analysis between CVL and the control groups were performed by one-way ANOVA using Tukey's multiple comparison test. Differences were considered significant when *P*<0.05. Data of shown in this study are representative of two independent vaccination' experiments, which presented similar results.

## Results

### Antigenicity of the *L. infantum* hypothetical protein, LiHyp1

**Figure 1:**
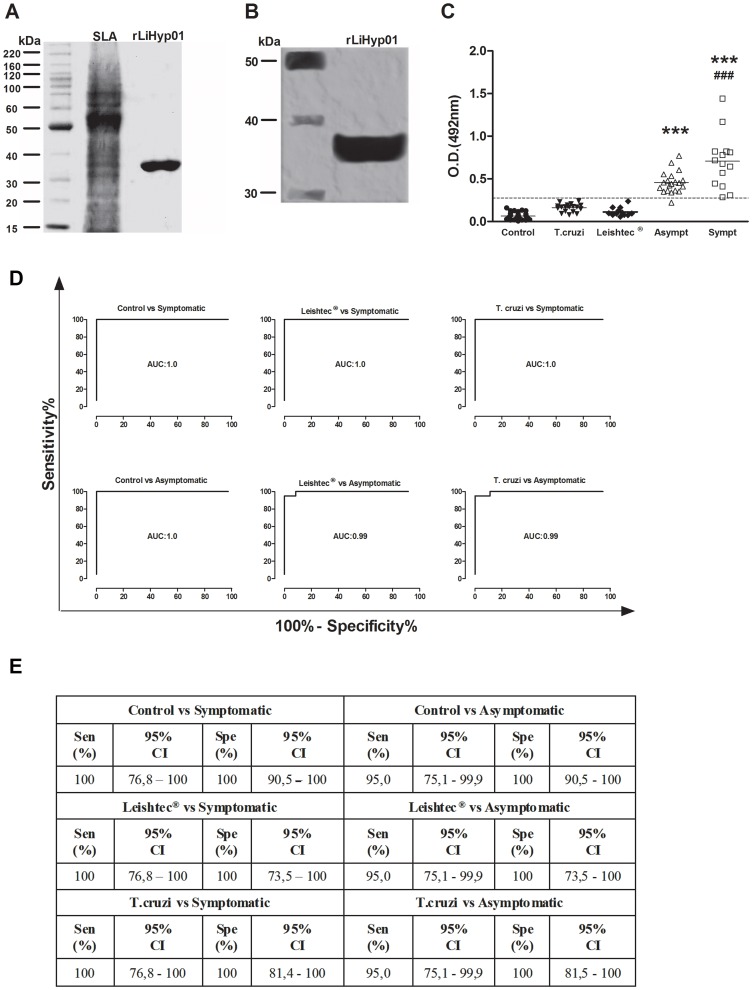
Antigenicity of rLiHyp1 protein against canine sera. Ten micrograms of *L. infantum* SLA and of the rLiHyp1 protein were electrophoresed on a SDS-PAGE 10% gel and stained with colloidal Coomassie Brilliant Blue G-250 (**A**). Western-Blot showing the reactivity against the purified protein from a pool of sera from dogs with asymptomatic VL. Gels and Western-Blot used were derived from three independent experiments, and one representative preparation was showed in this study (**B**). ELISA assays performed with rLiHyp1 (1 µg per well) against individual sera samples from healthy dogs (n = 37), *T. cruzi*-infected dogs (n = 18), Leish-Tec® vaccinated animals (n = 18); and asymptomatic (n = 19) and symptomatic (n = 15) *L. infantum*-infected dogs is showed (**C**). The OD values of each individual serum are shown. Cut-off value (dotted line) for negative and positive samples discrimination was selected by Receiver-Operating Characteristic (ROC) curves analysis as the lower OD value with a 100% of specificity. Statistically significant differences were obtained between CVL groups and other groups: *** *P<0.0001*; and between the symptomatic and asymptomatic groups: ^###^ *P*<0.0001, by one-way ANOVA using the Tukey's multiple comparison test. In order to analyze the diagnostic performance of rLiHyp1 against different sera samples, ROC curves were used to determine area under curve (AUC) (**D**), and sensitivity and specificity values (**E**).

In the present study, a putative member of the super-oxygenase family in *Leishmania* was fused as a recombinant protein to an N-terminal 6× histidine-tag and expressed in *E. coli*. The recombinant protein (rLiHyp1) was purified by nickel affinity chromatography (Fig. 1A), and tested for serodiagnosis of CVL. Initially, a pool of sera of asymptomatic dogs was able to recognize the rLiHyp1 by immunoblotting analysis, as seen in Fig. 1B.

Sera were individually tested in ELISA against rLiHyp1, and the results indicated that all sera samples of symptomatic dogs, and 18 out of 19 samples of asymptomatic CVL animals were able to recognize the recombinant protein. In contrast, antibodies from *T. cruzi*-infected, Leish-Tec® vaccinated or healthy dogs did not react with the rLiHyp1 protein (Fig. 1C). To determine the diagnostic performance of rLiHyp1 for CVL, Receiver-Operating Characteristic (ROC) curves were constructed to determine area under curve (AUC) and sensitivity and specificity values in the experiments. In the results, it was observed that the performance of rLiHyp1 proved to be highly effective in order to identify sera samples of symptomatic and asymptomatic CVL, and also to differentiate them in relation to the other sera samples employed in this study (Fig. 1D and 1E, respectively).

### Immunogenicity and protective efficacy of rLiHyp1 against *L. infantum*

**Figure 2:**
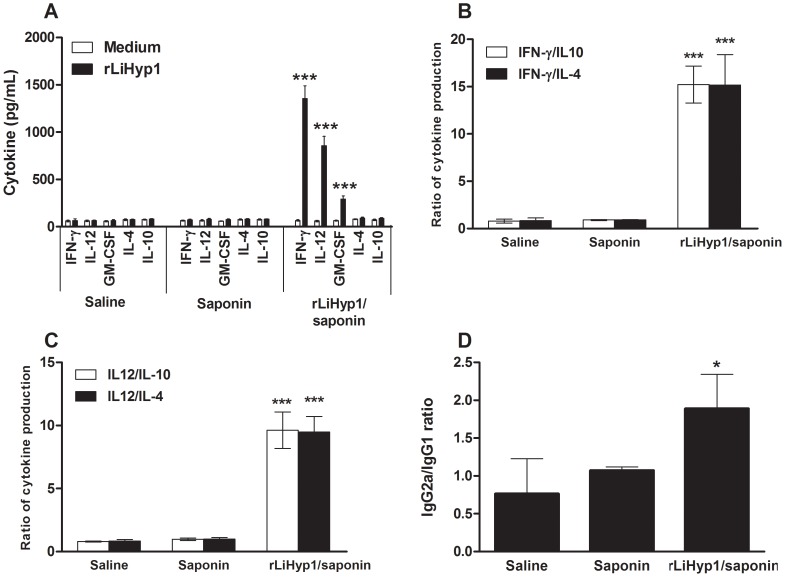
Cellular and humoral response induced in BALB/c mice by immunization with rLiHyp1 plus saponin. Single cells suspensions were obtained from the spleens of mice, four weeks after vaccination. Cells were non-stimulated (medium; background control) or stimulated with rLiHyp1 (20 µg mL^−1^) for 48 h at 37°C, 5% CO_2_. IFN-γ, IL-12, GM-CSF, IL-4, and IL-10 levels were measured in culture supernatants by capture ELISA (**A**). Each bar represents the mean ± standard deviation (SD) of data from four individual mice per group. Statistically significant differences in the IFN-γ, IL-12 and GM-CSF levels between the rLiHyp1 plus saponin group and control mice (saline and saponin groups) were observed (*** *P*<0.0001). The ratio between IFN-γ/IL-10 and IFN-γ/IL-4 levels (**B**); and between IL-12/IL-10 and IL-12/IL-4 levels (**C**) are also showed. Statistically significant differences in the ratios between the rLiHyp1 plus saponin group and control groups were observed (*** *P*<0.0001). The ratio between rLiHyp1-specific IgG1 and IgG2a antibodies was obtained for sera of each individual mouse within their respective vaccination group and statistically significant difference between the rLiHyp1 plus saponin group and control groups was also observed (* *P*<0.005) (**D**).

The immunogenicity of the rLiHyp1 was evaluated in BALB/c mice, 4 weeks after the last vaccine dose. Following *in vitro* stimulation with rLiHyp1, spleen cells from vaccinated mice significantly produced higher levels of IFN-γ, IL-12, and GM-CSF than those secreted by spleen cells from control mice (saline and saponin groups). No increase in IL-4 and IL-10 production could be observed in any experimental group, after stimulation with rLiHyp1 (Fig. 2A). The ratio between IFN-γ/IL-4 and IFN-γ/IL-10 levels; as well as between IL-12/IL-4 and IL-12/IL-10 levels showed that vaccinated animals presented an elevated Th1 immune response after rLiHyp1-stimulus (Fig. 2B and 2C, respectively). In addition, mice vaccinated with rLiHyp1 plus saponin presented an rLiHyp1-specific humoral response, with the predominance of IgG2a isotype (Fig. 2D).

**Figure 3:**
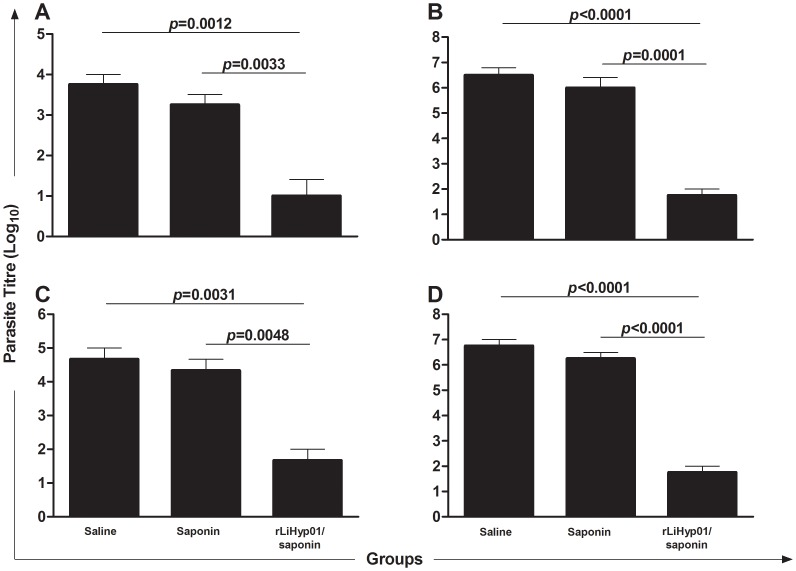
Protection of BALB/c mice vaccinated with rLiHyp1 plus saponin against *L. infantum*. Mice inoculated with saline, saponin, or rLiHyp1 plus saponin were subcutaneously infected with virulent 1×10^7^ stationary-phase promastigotes of *L. infantum*. The number of parasites in the liver (**A**), spleen (**B**), bone marrow (**C**), and paws' draining lymph nodes (**D**) was measured, 10 weeks after challenge by a limiting-dilution technique. Mean ± standard deviation (SD) of four mice in each group is shown. Statistically significant differences in the parasite load in all evaluated organs between the rLiHyp1 plus saponin group and control mice (saline and saponin groups) are showed (in numbers). Data shown in this study are representative of two independent experiments, which presented similar results.

Next, the present study analyzed whether the immunization with the rLiHyp1 plus saponin was able to induce protection against *L. infantum*. The infection was followed up over a 10-weeks period, when the parasite burden in the liver, spleen, BM, and dLN was determined. Significant reductions in the number of parasites were observed in the different evaluated organs of vaccinated mice, as compared with those that received only saline or saponin (Fig. 3). In this context, vaccinated mice with rLiHyp1 plus saponin showed significant reductions in the parasite load in liver (3.8- and 3.3-log reductions, Fig. 3A), spleen (3.7- and 3.5-log reductions, Fig. 3B), BM (3.0- and 3.0-log reductions, Fig. 3C), and dLN (3.9- and 3.6-log reductions, Fig. 3D), in comparison to the saline and saponin groups, respectively. Attempting to determine the influence of immunization with rLiHyp1 plus saponin on the *L. infantum* specific killing effectors functions in the spleen of infected mice, nitrite was assayed as an indicator of nitric oxide (NO) production in spleen cells. The nitrite production was significantly higher in mice vaccinated with rLiHyp1 plus saponin after stimulation with SLA, as compared to the control groups that produced minimum amounts of this product (data not shown).

### Cellular response elicited after L. infantum challenge infection

**Figure 4:**
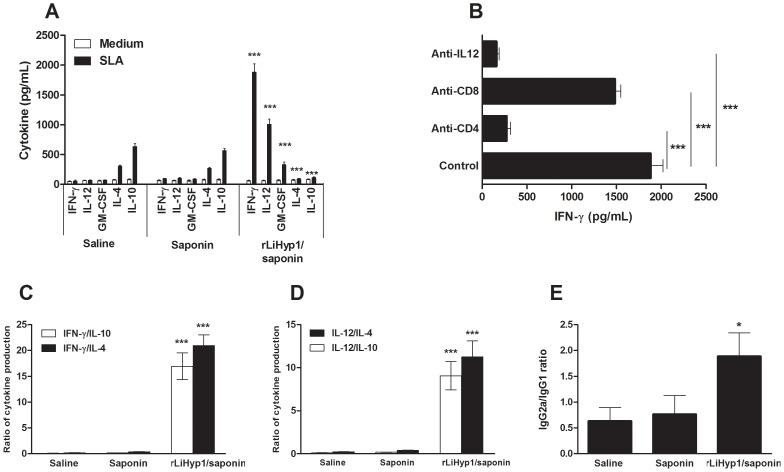
Analysis of the cellular and humoral response and of the involvement of IL-12, CD4 and CD8 T cells in the IFN-γ production after *L. infantum* challenge. Single cells suspensions were obtained from the spleens of mice, 10 weeks after infection. Cells were non-stimulated (medium; background control) or stimulated with *L. infantum* SLA (25 µg mL^−1^) for 48 h at 37°C, 5% CO_2_. Levels of IFN-γ, IL-12, GM-CSF, IL-4 and IL-10 were measured in culture supernatants by capture ELISA. Mean ± standard deviation (SD) of the cytokines levels determined in four individual mice per group is shown (**A**). Statistically significant differences between the rLiHyp1 plus saponin group and the control mice (saline and saponin groups) were observed (*** *P<0.0001*). The analysis of the involvement of IL-12 and CD4 and CD8 T cells in the IFN-γ production is showed (**B**). Levels of IFN-γ in the supernatants of spleen cells cultures stimulated with SLA, as explained above, in the absence (positive control) or in the presence of anti-IL-12, anti-CD4, or anti-CD8 monoclonal antibodies were measured. Statistically significant differences between non-treated control cells and cultures incubated with anti-CD4 and anti-IL-12 monoclonal antibodies were observed (*** *P*<0.0001). The ratio between IFN-γ/IL-10 and IFN-γ/IL-4 levels (**C**), and between IL-12/IL-10 and IL-12/IL-4 levels (**D**), are also showed. Statistically significant differences between the rLiHyp1 plus saponin group and the control groups were observed (***P<0.0001). The ratio between SLA-specific IgG1 and IgG2a antibodies levels were calculated for sera of each individual mouse within their respective vaccination group and statistically significant difference between the rLiHyp1 plus saponin group and the control groups was also observed (* *P*<0.005) (**E**).

The production of cytokines in the supernatants of spleen cells cultures stimulated with rLiHyp1 and SLA after challenge was analyzed to determine the immunological correlates of protection induced by rLiHyp1. The spleen cells from mice vaccinated with rLiHyp1 plus saponin produced higher levels of SLA-specific IFN-γ, IL-12 and GM-CSF cytokines than those secreted by spleen cells from control groups, 10 weeks after infection (Fig. 4A). In contrast, the SLA-driven production of IL-4 and IL-10 showed that vaccination with rLiHyp1 plus saponin induced no production of these cytokines in the vaccinated and infected animals.

The contribution of CD4 and CD8 T cells and the dependence of IL-12 production for the SLA-specific IFN-γ response from the spleen cells of mice immunized with rLiHyp1 plus saponin and challenged with *L. infantum* were evaluated. The IFN-γ production was completely suppressed using anti-IL-12 or anti-CD4 monoclonal antibodies in the spleen cells cultures (Fig. 4B). The addition of anti-CD8 antibodies to the cultures also decreased the production of IFN-γ, as compared to the cell cultures without treatment (1.881±139 pg/mL before, and 1.533±110 pg/mL after including anti-CD8 antibodies); however, the production of this cytokine proved to be higher than that produced by the use of the anti-CD4 monoclonal antibody.

As observed before challenge, the ratio between IFN-γ/IL-4 and IFN-γ/IL-10, and between IL-12/IL-4 and IL-12/IL-10 indicated that vaccinated mice developed a specific Th1 immune response, which was maintained after infection in these animals (Fig. 4C and 4D, respectively). In this study, very low levels of anti-SLA antibodies could be observed in the sera of all mice groups challenged with *L. infantum*. However, it was possible to detect that vaccinated and infected mice presented SLA-specific IgG2a antibodies that were significantly higher than the obtained IgG1 levels (Fig. 4E).

## Discussion

Different *Leishmania* proteins with antigenic properties were recently identified by an immunoproteomic approach applied to *L. infantum* promastigotes and amastigote-like proteic extracts [18], including hypothetical proteins of the parasites. The fact that antibodies present in the sera of infected dogs recognized these hypothetical proteins indicates that they are expressed by parasites during active infection, and are antigenic to the host's immune system. In this context, the DNA encoding one of these *Leishmania* hypothetical proteins, which was specifically recognized by antibodies in the amastigote-like antigenic extracts, was cloned and expressed in *E. coli* and tested for its antigenicity and prophylactic properties. Immunoblotting and ELISA analyses demonstrated that the recombinant LiHyp1 protein (rLiHyp1) was specifically recognized by antibodies present in the sera of dogs with symptomatic and asymptomatic VL, yet it presented no cross-reactivity with the sera of dogs vaccinated with a Brazilian recombinant vaccine, Leish-Tec®, or with animals experimentally infected with *T. cruzi*, demonstrating, besides antigenicity its potential for improvement of CVL serodiagnosis. Dogs are also reservoirs for *T. cruzi* parasites in endemic areas for VL transmission in Brazil, and seroprevalences of anti-*T. cruzi* antibodies, which may cross react with *Leishmania* antigens, of 21.9% and up to 57.0% have been reported [19], [20].

In a previous study, it was demonstrated that sera from dogs naturally infected with *L. infantum* displayed reactivity with *Leishmania* ribosomal proteins (LRP) through Western-Blot analysis. A comparison between LRP and SLA showed that LRP had a similar sensitivity in ELISA, but higher specificity than the SLA-based assays in the diagnosis of CVL [21]. However, the technical purification of LRP is complex and labor-intensive. In contrast, the production of recombinant proteins is less complex and allows obtain large amounts of proteins, when compared to production of semi-purified extracts of the parasites (like LRP). In this context, the use of the rLiHyp1 protein in the serodiagnosis of CVL is attractive.

Amastigote antigens have been far less tested as vaccine candidates against VL [15]. Therefore, a vaccine that is able to elicit immune responses against intracellular amastigotes of *Leishmania* may present advantages not only for prophylactic, but also for therapeutic vaccines. In this context, the immunization with rLiHyp1 plus saponin was able to induce a predominant Th1 immune response, which was characterized by an *in vitro* rLiHyp1-specific production of IFN-γ, IL-12 and GM-CSF, combined with the presence of very low levels of IL-4 and IL-10. After infection, mice immunized with rLiHyp1 plus saponin, when compared to control groups, displayed significant reductions of the number of parasites in all evaluated organs (liver, spleen, BM, and dLN), which correlated a specific rLiHyp1- and SLA-dependent IFN-γ production in the spleen, one of the main cytokines implicated in the acquired immunity against infection with *Leishmania* [22], [23], [24]. The CD4 T cells proved to be the major source of IFN-γ in the protected mice, since depletion of these cells in cultures of spleen cells stimulated with SLA significantly abrogated this response. Similarly, in the vaccinated mice, IFN-γ production proved to be IL-12-dependent. Although previous reports have shown that the activation of both CD4 and CD8 T cells subsets may be important for the killing of parasites in mice vaccinated with different parasite recombinant antigens [25], [26], the present study's data suggest that CD8 T cells may contribute in a less extension to the induction of IFN-γ mediated response elicited by the rLiHyp1 plus saponin formulation. Besides production of IFN-γ, these cells may contribute to infection control by their direct cytotoxic effect on infected cells, as previously demonstrated in other experimental conditions [24]. Altogether, the present study indicates that immunization with rLiHyp1 plus saponin primed BALB/c mice for an rLiHyp1-specific Th1 response that was sustained after *L. infantum* infection challenge.

The present study also showed that the protection in BALB/c mice against *L. infantum* is associated with a significant decrease in the production of macrophage deactivating cytokines, like IL-4 and IL-10. Very low levels of *Leishmania*-specific IL-10 were detected after the stimulation of spleen cells from vaccinated mice, 10 weeks after infection. In contrast, spleen cells from both control mice groups showed a significantly higher production of this cytokine. Indeed, control of the parasite-mediated IL-10 response in vaccinated mice may be critical for protection, since this cytokine is considered to be the most important factor for VL progression after infection with viscerotropic *Leishmania* species in IL-10 deficient mice [12],[27],[28], or in mice treated with an anti-IL-10 receptor antibody [29]. In BALB/c mice, the IL-4-dependent production of IgG1 antibodies is associated with disease progression due to some *Leishmania* species, including *L. amazonensis* [30], but it is not confirmed in *L. infantum* or *L. donovani* [31],[32]. Nonetheless, in BALB/c mice vaccinated with recombinant A2 protein or LRP plus saponin, the protection against cutaneous or visceral leishmaniasis have been also correlated with a decrease in *Leishmania*-specific IL-4 and IL-10 mediated response [12], [17], [22], [33].

Spleen cells from vaccinated mice, as compared to the control groups, produced higher levels of rLiHyp1- and SLA-specific GM-CSF, a cytokine related with macrophage activation and resistance in murine models against different intracellular pathogens, including *L. major* [34], *L. donovani* [35], and *L. chagasi* ( = *L. infantum*) [12]. It has also been shown that the immunization of humans with a crude *Leishmania* antigenic preparation using this cytokine as an adjuvant commonly induces a parasite-specific Th1 response [36], and that the administration of a therapeutic vaccine containing some *Leishmania* antigens plus GM-CSF could be correlated with the cure of lesions in the muco-cutaneous leishmaniasis [37].

A critical aspect for *Leishmania* vaccines development refers to the pre-clinical model chosen for initial screening of vaccine candidates. Although sand fly transmitted infection in hamsters more closely resemble the natural transmission and the human disease, this infection model requires specific laboratory conditions and trained personnel staff, which are not widely available, hindering its general use as a first step for testing vaccine efficacy against VL [38]. In contrast, BALB/c mice infected with *L. donovani* or *L. infantum* is one the most widely studied murine models for VL, and is therefore naturally selected over other models for this purpose [17], [39], [40], [41]. Murine models have also allowed the characterization of the immune mechanisms required to develop organ-specific immune response against *Leishmania* [41], [42]. Therefore, the evaluation of the parasite burden in different organs is an important marker of vaccine efficacy against VL in these models. In a recent study, it was demonstrated that the subcutaneous route of inoculation of *L. infantum* in BALB/c mice induces a faster infection development in the animals and higher parasite burden in different tissues as compared to the intravenous challenge [41]. In this context, the subcutaneous route was selected to evaluate the efficacy of rLiHyp1 plus saponin vaccine against *L. infantum*. In addition, in comparative studies, it was found that protection afforded by vaccination might be improved in animals challenged by intradermal/subcutaneous route as compared to those receiving an intravenous challenge [43], [44]. Nevertheless, additional studies may well be carried out in order to extend the observations present herein of the protective efficacy of rLiHyp1 plus saponin vaccination to other infection models and experimental conditions.

In conclusion, the present study's data indicated that a *Leishmania* amastigote-specific protein, member of the super-oxygenase family, LiHyp1, is antigenic in the CVL, and also conferred protection in BALB/c mice against *L. infantum*. Protection correlated with the CD4 T cells response characterized by high IFN-γ, IL-12, and GM-CSF, and low IL-4 and IL-10 levels. Therefore, the LiHyp1 protein constitutes a new and promising antigen candidate for serodiagnosis and vaccine development against VL.
