# Correction: Antigenicity and Protective Efficacy of a *Leishmania* Amastigote-specific Protein, Member of the Super-oxygenase Family, against Visceral Leishmaniasis

**DOI:** 10.1371/annotation/427655ac-c278-41f5-95ca-6279c562752f

**Published:** 2013-04-16

**Authors:** Vivian T. Martins, Miguel A. Chávez-Fumagalli, Lourena E. Costa, Adriana M. C. Canavaci, Paula S. Lage, Daniela P. Lage, Mariana C. Duarte, Diogo G. Valadares, Rubens D. M. Magalhães, Tatiana G. Ribeiro, Ronaldo A. P. Nagem, Wanderson D. DaRocha, Wiliam C. B. Régis, Manuel Soto, Eduardo A. F. Coelho, Ana Paula Fernandes, Carlos A. P. Tavares

The author listed as Adriana M.C.C. Martins should read as Adriana M.C. Canavaci. The citation should read as follows:

Martins VT, Chávez-Fumagalli MA, Costa LE, Canavaci AMC, Lage PS, et al. (2013) Antigenicity and Protective Efficacy of a Leishmania Amastigote-specific Protein, Member of the Super-oxygenase Family, against Visceral Leishmaniasis. PLoS Negl Trop Dis 7(3): e2148. doi:10.1371/journal.pntd.0002148

